# Case Report: One heart with two lobes: a rare infantile congenital giant left atrial appendage aneurysm

**DOI:** 10.3389/fped.2023.1302182

**Published:** 2023-11-20

**Authors:** Yu-Jen Chan, Nguyen Thi Ly Ly, Nguyen Minh Hai, Sheng-Ling Jan

**Affiliations:** ^1^ Department of Pediatrics, Da Chien Hospital, Miaoli, Taiwan; ^2^ Division of Pediatric Cardiology, Dong Nai Children’s Hospital, Bien Hoa, Vietnam; ^3^ Division of Pediatric Cardiology, Nhi Dong No1 Hospital, Ho Chi Minh, Vietnam; ^4^ Division of Pediatric Cardiology, Children’s Medical Center, Taichung Veterans General Hospital, Taichung, Taiwan; ^5^ Department of Post-Baccalaureate Medicine, College of Medicine, National Chung Hsing University, Taichung, Taiwan; ^6^ Department of Pediatrics, School of Medicine, Kaohsiung Medical University, Kaohsiung, Taiwan

**Keywords:** left atrial appendage aneurysm, cardiomegaly, dextroposition of heart, congenital heart disease, surgical repair

## Abstract

Left atrial appendage aneurysm (LAAA) is an extremely rare congenital heart abnormality, with varying degrees of symptoms, ranging from asymptomatic to arrhythmia, thromboembolic event or airway obstruction. Most infantile cases were incidentally found by echocardiography. Contrast-enhanced chest tomography can confirm the diagnosis and inform surgical plan. We describe an asymptomatic young female infant who had a unique extreme cardiomegaly on a chest x-ray and received surgical aneurysmectomy. Her heart was restored to a normal cardiac size after the heart surgery.

## Introduction

When significant cardiomegaly is observed on chest radiography in young infants, several potential etiologies may be considered, including the extremely rare left atrial appendage aneurysm (LAAA) (1). LAAA is a very rare congenital abnormality characterized by abnormal aneurysm formation connecting to the left atrial appendage, which may be asymptomatic, or may present with arrhythmia, thromboembolic event or airway obstruction (2–4). While LAAA has been reported in teenagers and adults, occurrence in infants is rare (5, 6). We present an unusual case of a baby girl diagnosed with a giant left atrial appendage aneurysm, which was managed by aneurysmal resection using a cardiopulmonary bypass.

## Case report

**Figure 1:**
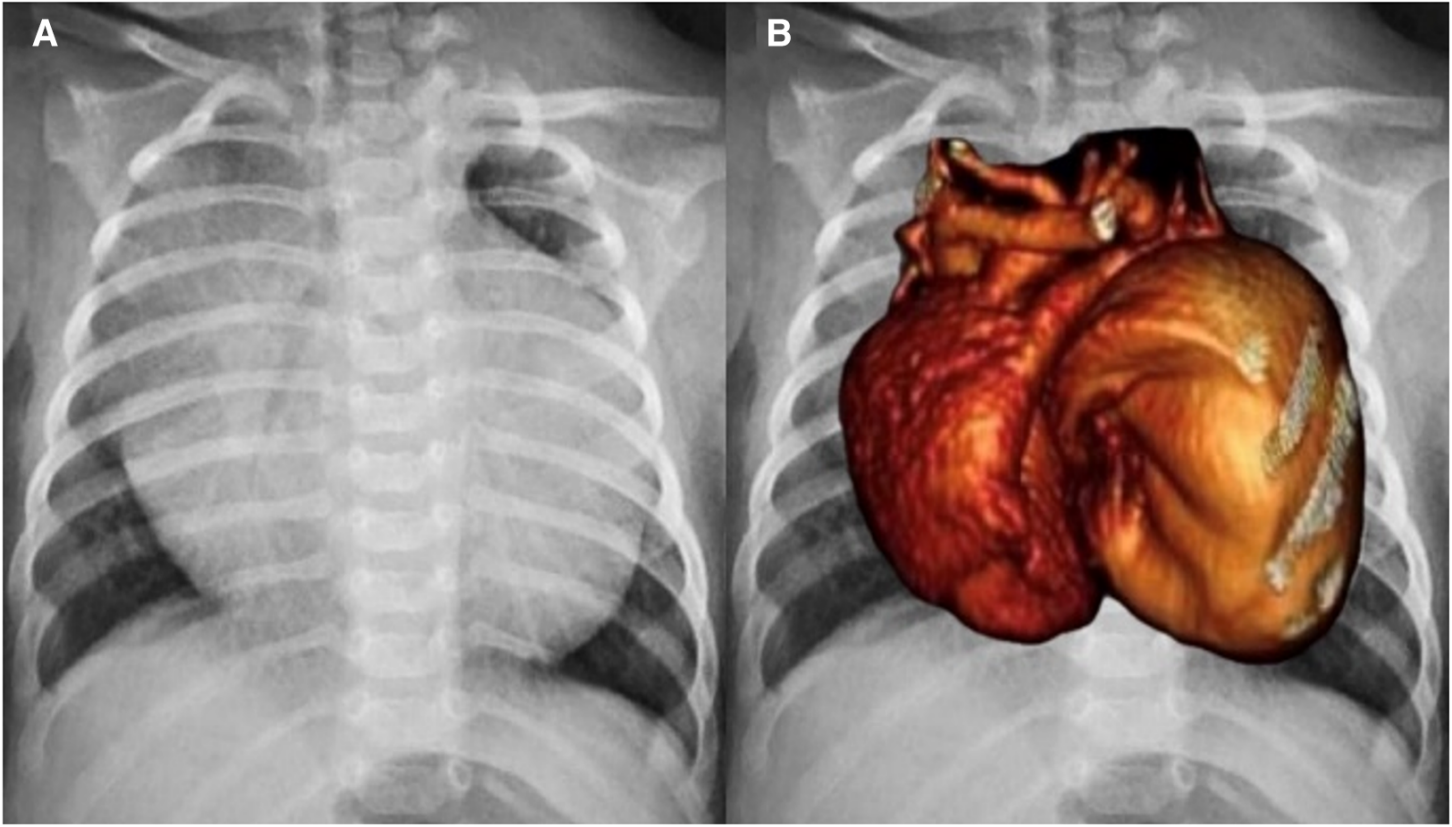
(**A**) Chest radiograph revealed extreme cardiomegaly (cardiothoracic ratio: 0.82). (**B**) Cardiac silhouette of one heart with two lobes was shown on the chest radiograph and was merged with a 3-dimensional multidetector-computed tomography.

**Figure 2:**
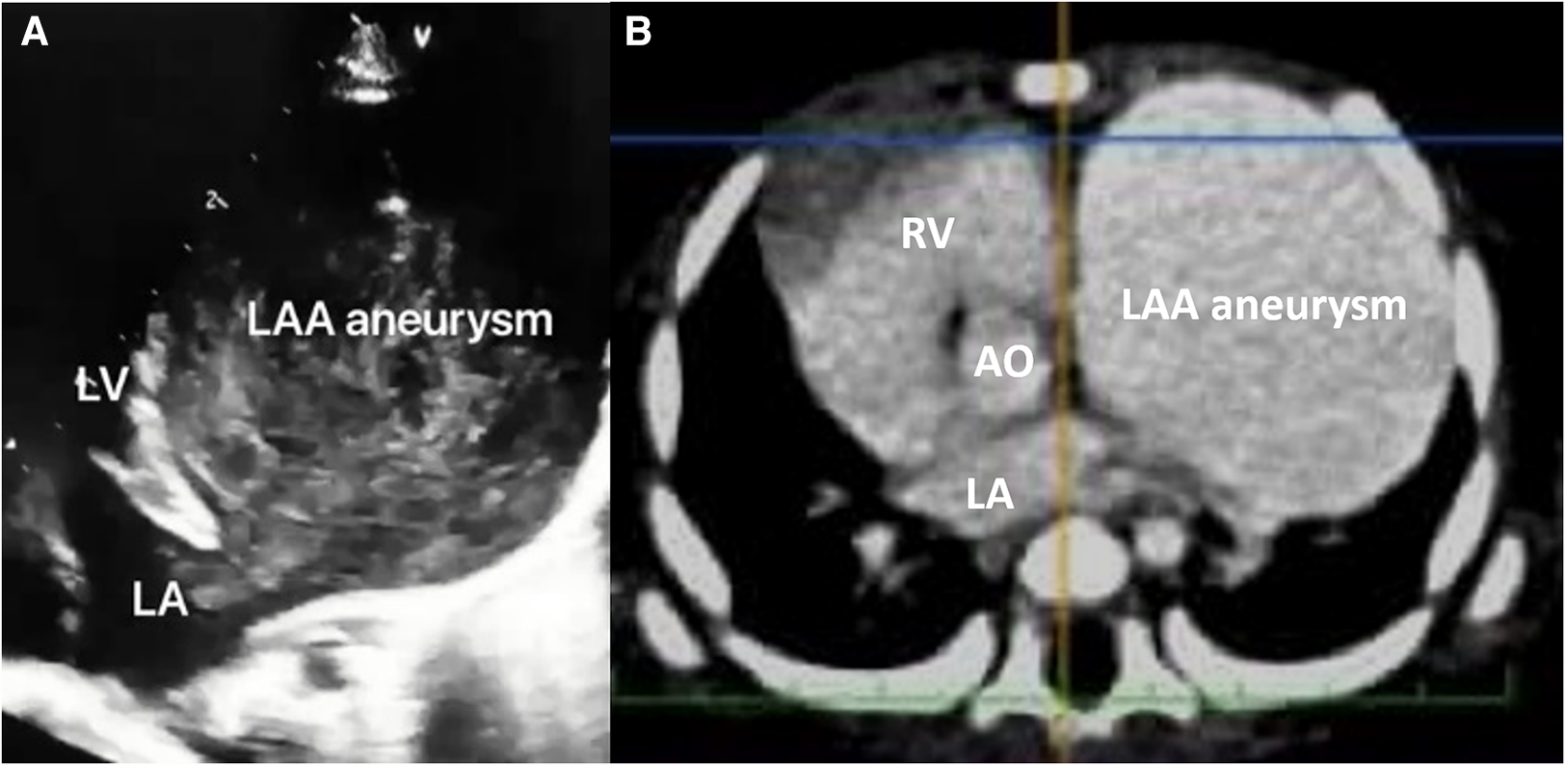
(**A**) Apical four-chamber view in transthoracic echocardiography revealed a giant aneurysm formation connecting to the left atrium, and (**B**) contrast-enhanced computed tomography demonstrated a huge cystic lesion (filled with contrast), which made the heart deviate to the right side. AO, aorta, LA, left atrium, LV, left ventricle, RV, right ventricle, LAA, left atrial appendage.

**Figure 3:**
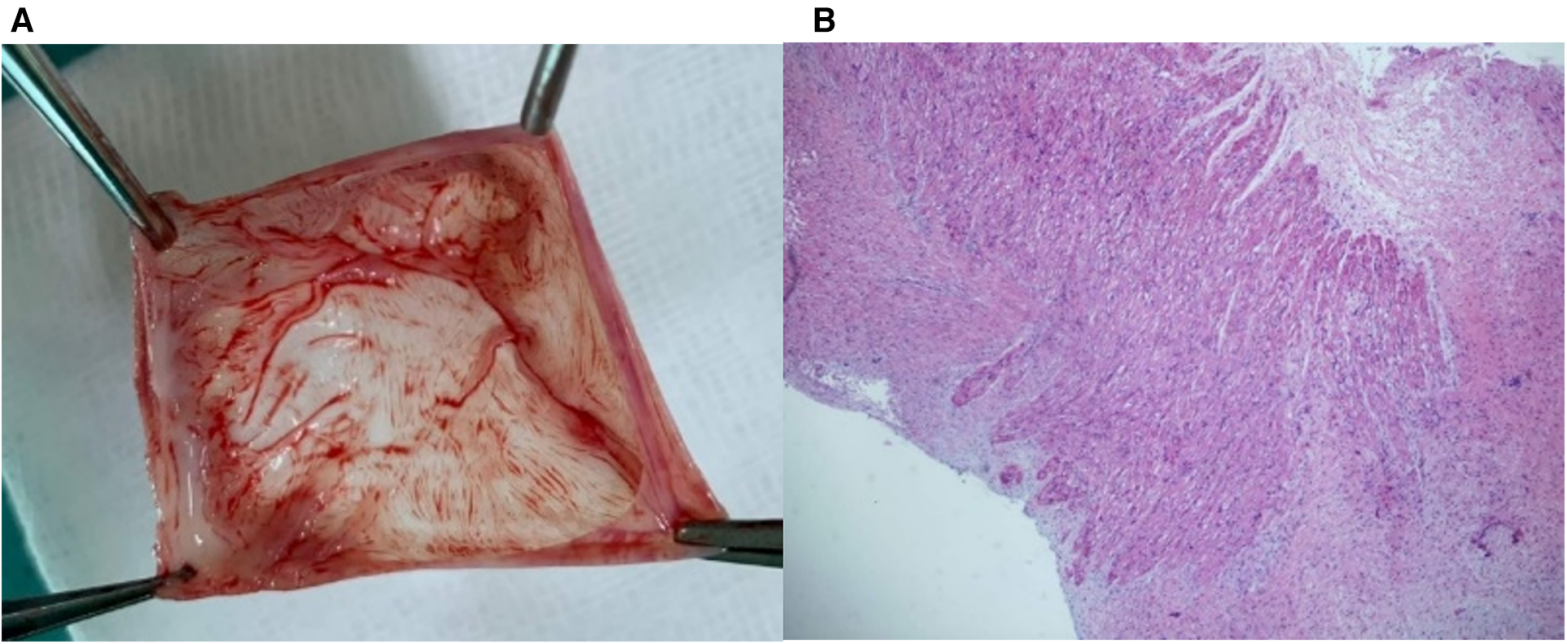
(**A**) A 3.5 × 2 × 1 cm cyst was resected from the left atrium. (**B**) Pathology showed fibrous and smooth muscle tissue content, consistent with left atrial appendage aneurysm.

**Figure 4:**
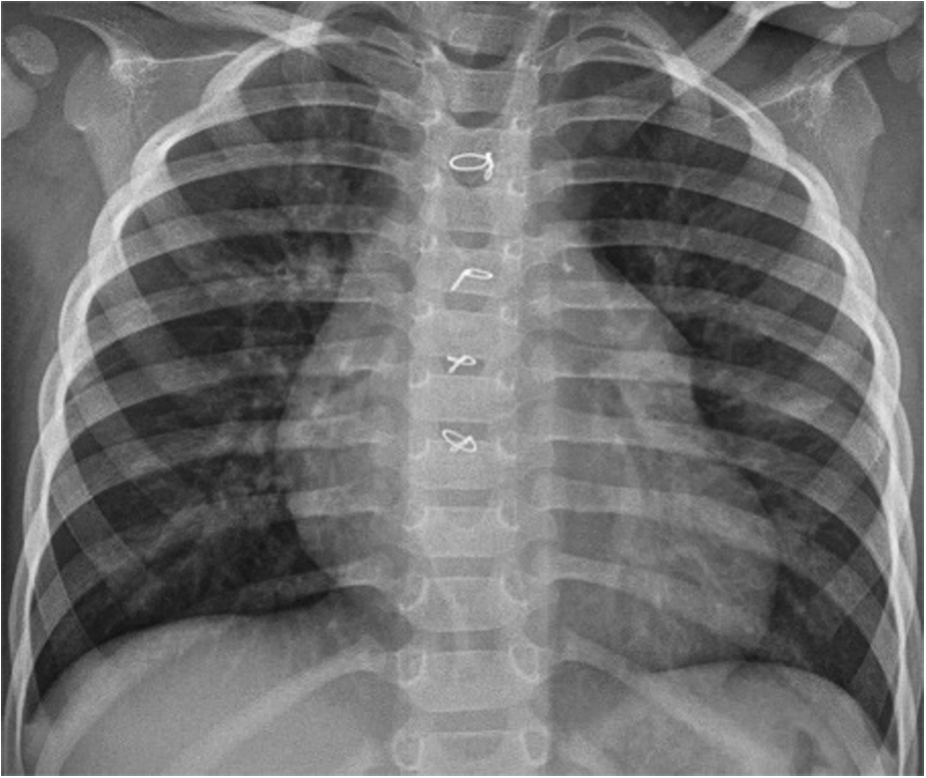
A post-aneurysmectomy chest radiograph showed a normal-sized heart with a normal silhouette of the left atrial appendage.

A full-term female infant with a birth weight of 2,700 g was born via normal vaginal delivery and received the Baccilus Calmette-Guerin (BCG) vaccine after birth. At approximately 1.5 months of age, the presence of a neck mass prompted a visit to the pediatric outpatient department. The chest x-ray incidentally revealed significant cardiac enlargement (Figure 1A). Physical examination found a soft, non-tender lymph node 2 cm in diameter above the left clavicle, but there were no limb edema, hepatosplenomegaly, cardiac murmurs, or signs of growth retardation. The patient was subsequently referred to the pediatric cardiology clinic, where an echocardiography showed a giant cystic lesion (41 × 47 mm) connecting to the left atrium, consistent with a LAAA (Figure 2A). Further imaging with 3-dimensional multidetector-computed tomography (MDCT) confirmed the diagnosis (Figures 1B, 2B). Surgical intervention was recommended for LAAA resection. Normal *P* wave axis with right axis deviation and absent *R* wave progression in the precordial leads were noted in the preoperative electrocardiography, which may be a clue for dextroposition of the heart. The surgical approach involved a median sternotomy and a cardiopulmonary bypass, without hypothermia. The left atrial appendage was opened, and the contained blood was aspirated, causing the aneurysm to deflate. The LAAA was then resected through the opening. The lymph node over the neck was removed. The surgery was successful without any complications. The patient was discharged 7 days after the heart surgery. The pathology of the aneurysm revealed a 3.5 cm × 2.0 cm × 1.0 cm cystic mass containing fibrous and smooth muscle tissue (Figure 3). A post-aneurysmectomy chest radiograph showed a normal-sized heart with a normal silhouette of the left heart border (Figure 4). The patient remained asymptomatic with normal physical exam and echocardiography at her postoperative follow-ups.

## Discussion

LAAA in infants pose unique challenges in terms of diagnosis and management. In contrast to adult LAAA case, which may be presented with greatly enlarged left heart border in chest x-ray (7). Our baby girl represented with significant cardiomegaly and double lobbed heart which consisted with a giant LAAA and dextro-positioned heart. The variable morphology has been discussed in several studies, and different shapes have been observed in imaging examinations depending on factors such as the patient's age, the presence of thrombus, and the presence of other associated heart diseases (8, 9). When significant cardiomegaly is observed on chest radiography in young infants, several potential etiologies may be considered, including congenital heart disease, cardiomyopathy, perinatal myocarditis, inborn errors of metabolism, such as glycogen storage disease, lysosomal storage disease, and mitochondrial disease, heart tumors, and more rarely, LAAA (1). It is important to note that extreme cardiomegaly in such infants is a serious condition that requires immediate medical attention and thorough evaluation by a pediatric cardiologist (1). When a chest radiography alone is not sufficient to establish a definitive diagnosis, further evaluation, including clinical evaluation, electrocardiography, echocardiography, cardiac MDCT or magnetic resonance, and genetic testing, is necessary to determine the underlying cause and guide appropriate management.

While the exact etiology of LAAA remains unclear, congenital dysplasia of the musculi pectinati is believed to play a significant role (10). Diagnostic imaging, including echocardiography and computed tomography, plays a crucial role in confirming the diagnosis and assessing the extent of the aneurysm (11, 12). Fetal echocardiography could even detect LAAA if the lesion is large enough (13, 14). The characteristic cystic appearance and connection to the left atrium helps to differentiate it from other extracardiac mass or abnormalities. Additionally, these imaging modalities aid in surgical planning. Surgical intervention is necessary due to the significant size of the aneurysm and the potential risk of complications, such as thromboembolism and arrhythmia. Aneurysmal resection through median sternotomy or minimally invasive endoscopic technique both have favorable outcomes (5, 15). Concomitant atrial fibrillation ablation (e.g., Maze procedure) can be considered in adult patients with higher risk of atrial fibrillation (6). Transcatheter occlusion with an LAA occluder has also been recently reported in a selected adult patient (16).

## Conclusion

This case highlights the rarity of LAAAs in infants and emphasizes the importance of a multidisciplinary approach involving pediatric cardiology, radiology, and cardiothoracic surgery in the diagnosis and management. Early identification and timely surgical intervention are crucial in preventing potential complications associated with LAAAs, particularly in young infants. Further research is needed to understand the underlying etiology and long-term outcomes of this condition in the pediatric population.

## Data Availability

The original contributions presented in the study are included in the article/**Supplementary Material**, further inquiries can be directed to the corresponding author.
